# Early maturation and distinct tau pathology in induced pluripotent stem cell-derived neurons from patients with *MAPT* mutations

**DOI:** 10.1093/brain/awv222

**Published:** 2015-07-28

**Authors:** Mariangela Iovino, Sylvia Agathou, Ana González-Rueda, Martin Del Castillo Velasco-Herrera, Barbara Borroni, Antonella Alberici, Timothy Lynch, Sean O’Dowd, Imbisaat Geti, Daniel Gaffney, Ludovic Vallier, Ole Paulsen, Ragnhildur Thóra Káradóttir, Maria Grazia Spillantini

**Affiliations:** 1 Department of Clinical Neurosciences, Clifford Allbutt Building, University of Cambridge, Cambridge, UK; 2 Wellcome-Trust Medical Research Council Stem Cell Institute and Department of Veterinary Medicine, University of Cambridge, Cambridge, UK; 3 Department of Physiology, Development and Neuroscience, Physiological Laboratory, University of Cambridge, Cambridge, UK; 4 Wellcome Trust Sanger Institute, Wellcome Trust Genome Campus, Hinxton, Cambridge, UK; 5 Department of Neurological Sciences, University of Brescia, Brescia, Italy; 6 Dublin Neurological Institute, Mater Misericordiae University Hospital and Conway Institute of Biomolecular and Biomedical Research, University College Dublin, Dublin Ireland; 7 Wellcome Trust-Medical Research Council Stem Cell Institute, Anne McLaren Laboratory, Department of Surgery, University of Cambridge, Cambridge UK

**Keywords:** FTDP-17T, microtubule-associated protein tau, neurofibrillary tangles, IPSC-derived neurons, *MAPT* gene mutations

## Abstract

Tauopathies, such as Alzheimer’s disease, some cases of frontotemporal dementia, corticobasal degeneration and progressive supranuclear palsy, are characterized by aggregates of the microtubule-associated protein tau, which are linked to neuronal death and disease development and can be caused by mutations in the *MAPT* gene. Six tau isoforms are present in the adult human brain and they differ by the presence of 3(3R) or 4(4R) C-terminal repeats. Only the shortest 3R isoform is present in foetal brain. *MAPT* mutations found in human disease affect tau binding to microtubules or the 3R:4R isoform ratio by altering exon 10 splicing. We have differentiated neurons from induced pluripotent stem cells derived from fibroblasts of controls and patients with N279K and P301L *MAPT* mutations. Induced pluripotent stem cell-derived neurons recapitulate developmental tau expression, showing the adult brain tau isoforms after several months in culture. Both N279K and P301L neurons exhibit earlier electrophysiological maturation and altered mitochondrial transport compared to controls. Specifically, the N279K neurons show abnormally premature developmental 4R tau expression, including changes in the 3R:4R isoform ratio and AT100-hyperphosphorylated tau aggregates, while P301L neurons are characterized by contorted processes with varicosity-like structures, some containing both alpha-synuclein and 4R tau. The previously unreported faster maturation of *MAPT* mutant human neurons, the developmental expression of 4R tau and the morphological alterations may contribute to disease development.

## Introduction

Alzheimer’s disease, progressive supranuclear palsy, corticobasal degeneration and several other neurodegenerative diseases are characterized by the presence of intracellular aggregates of microtubule-associated protein tau leading to their grouping under the name of tauopathies (Spillantini and Goedert, 2013). In the human adult brain, six tau isoforms are expressed that differ by the presence of 3(3R) or 4(4R) microtubule-binding domains in the carboxy-terminal part of the protein and by containing 0, 29 or 58 amino acid inserts in the amino-terminal part (Goedert *et al.*, 1989*a*). Tau expression is developmentally regulated and only the shortest tau isoform with 3R and no amino-terminal inserts is found in foetal brain, with all six isoforms appearing a few weeks after birth (Goedert *et al.*, 1989*b*).

The relevance of tau for neurodegeneration has been confirmed by the identification of mutations in the *MAPT* gene in cases with familial frontotemporal dementia and parkinsonism linked to chromosome 17 (FTDP-17T). So far more than 55 mutations have been identified in *MAPT* that cause the autosomal dominant disease (Ghetti *et al.*, 2015). The toxic mechanisms attributed to *MAPT* mutations vary, with some, like the P301L mutation in exon 10, predicted to affect microtubule-binding while others, like the N279K mutation, the silent mutations, and splicing mutations, having an effect on tau mRNA splicing, altering the expression of exon 10 that encodes the fourth repeat, leading to a change in the ratio of 3R:4R tau expression which is usually equal (Spillantini and Goedert, 2013). Clinically, FTDP-17T cases present with various phenotypes, the most common being frontotemporal dementia and parkinsonism, but some cases have also been defined as progressive supranuclear palsy, corticobasal degeneration and Alzheimer’s disease (Ghetti *et al.*, 2015). Where investigated, brains from FTDP-17T patients have shown tau aggregates with features similar to sporadic diseases with tau pathology. The mechanism of tau toxicity that leads to neurodegeneration is still unclear.

Studies of tauopathies have mainly relied on post-mortem human samples or animal models that often do not express all six adult human brain tau isoforms. Mice, for example, only express tau isoforms with 4R in adult brain. Therefore, the identification of a human neuronal system in which to investigate the effects of *MAPT* mutations and to test compounds for the treatment of tauopathies is desirable. Here we show that human induced pluripotent stem cell (IPSC)-derived neurons recapitulate the developmental pattern of brain tau, expressing initially the shortest 3R tau and later, after a few months in culture, both 3R and 4R adult brain tau isoforms. Furthermore, we show that two different mutations in *MAPT,* N279K and P301L, cause both common and specific phenotypes. In common, they show earlier neuronal maturation and altered anterograde mitochondrial axonal transport. Specifically, the P301L *MAPT* mutation shows thicker processes with varicosities some of which contain 4R tau and alpha-synuclein and abnormal mitochondrial retrograde transport. The N279K mutation, instead, presents specifically abnormal developmental expression of 4R tau, an imbalance in the 3R:4R tau isoform ratio and AT100^+ve^ hyperphosphorylated tau aggregates. The previously unreported developmental expression of 4R tau in N279K neurons, the varicosity-like structures in the P301L neurons, and the common earlier maturation phenotype may critically contribute to the pathogenesis of frontotemporal dementia with *MAPT* mutations.

## Material and methods

### Ethical approvals

Approval for ‘Generation of patient-specific stem cells for research in neurodegenerative disorders of the CNS’ was granted by the Hertfordshire Research Ethics Committee (ref n° 09/H0311/88).

Handling of human tissue was according to the UK Human Tissue Act 2006. The work on human tissue was covered by Cambridge LREC ethical approval (ref n° 09/40).

### Generation and culture of human IPSCs

**Figure 1:**
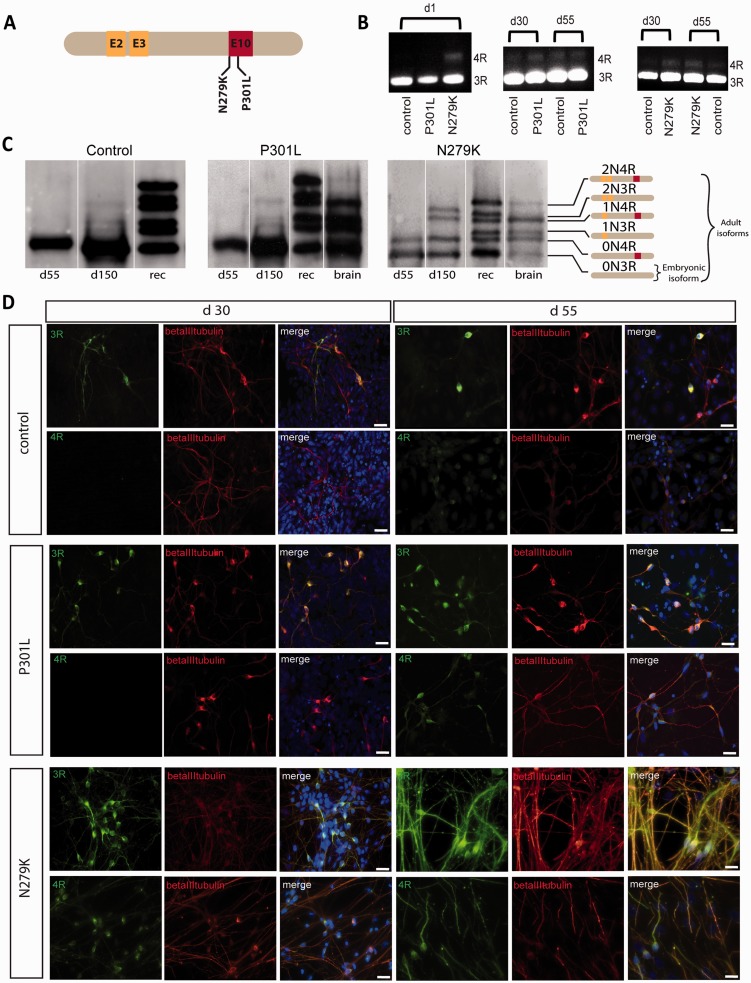
**Tau isoform expression in IPSC-derived neurons.** (**A**) Schematic diagram showing the position of the N279K and P301L mutations in the longest human brain tau isoform 2N4R. (**B**) Semiquantitative RT-PCR for 3R and 4R tau isoforms in control, P301L and N279K IPSCs at Day 1 (d1) shows that N279K cells already express 4R tau mRNA at the beginning of neuronal induction. Control and P301L cells express 4R tau mRNA at Day 30 at the neuronal precursor stage. Expression of 4R tau mRNA in N279K cells does not change significantly between Days 30 (d30) and 55 (d55). (**C**) Western blot analysis of soluble tau extracted with perchloric acid and treated with alkaline phosphatase in control, P301L- and N279K- derived neurons at Days 55 and 150 and in post-mortem human brain samples of the patient with the P301L mutation and a patient with the N279K mutation. Control and P301L neurons at Day 55 show the shortest 0N3R tau isoform while N279K neurons express both 3R and 4R tau isoforms. At Day 150 both P301L and N279K neurons express the six tau isoforms that are present in brain extracts of the patients carrying the respective mutations. Recombinant tau protein (Rec) is used as a tau isoform size control. In each panel lanes are from the same blot. (**D**) Immunocytochemistry for 3R and 4R tau isoforms at Days 30 and 55. In control and P301L neurons 3R tau is already present at Day 30, while 4R tau is visible in the cell bodies at Day 55. In N279K neurons both 3R and 4R tau isoforms are already present at Day 30 with 3R tau localized in both cell bodies and processes while 4R tau is in cell bodies. At Day 55 in N279K neurons 3R and 4R tau are equally distributed in cell bodies and processes while in P301L and control neurons 4R tau is still mainly in cell bodies. Scale bars = 25 µm.

Skin biopsies (3 mm punch biopsies) were obtained from two patients with the *MAPT* gene P301L mutation (Mirra *et al.*, 1999; Alberici *et al.*, 2004), which alters tau microtubule-binding, and one patient with the N279K mutation (O’Dowd *et al.*, 2012), which alters an exonic splicing regulatory element, affecting tau mRNA splicing and the 3R:4R tau isoform ratio (Clark *et al.*, 1998; Hasegawa *et al.*, 1999; Slowinski *et al.*, 2007; O’Dowd *et al.*, 2012) (Fig. 1A). Fibroblasts from these skin biopsies were produced at the hIPSC core facility of the MRC Laboratory of Regenerative Medicine at the University of Cambridge, Cambridge, UK. Fibroblasts from two other patients with the N279K mutation were obtained from the NINDS Coriell Biorepository (https://www.coriell.org/). The characteristics of the subjects providing fibroblasts for IPSC generation are reported in Table 1. All fibroblasts were derived into IPSCs at the MRC Laboratory of Regenerative Medicine including IPSCs from three control subjects (Vallier *et al.*, 2009), which were provided by Dr L. Vallier (Table 1). Chromosomal rearrangements were identified by karyotyping the IPSCs from one patient with the N279K lines and one of the control subjects and although the *MAPT* locus or Ch17 were not directly involved in the genomic alterations, these IPSC lines were removed from further studies (Table 1).

**Table 1:** Characteristics of subjects providing fibroblasts for IPSC derivation and brain tissue

Patient samples	Age	Sex	Diagnosis	Karyotype	Brain tissue for western blotting or immunohistochemistry	Ref case
Control (I)	28	Female	Healthy	✓		
Control (II) ATCC/ CRL 2429	Newborn	Male	Healthy	✓		Vallier *et al.* (2009); Shi *et al.* (2012)
Control(III)^a^	49	Male	Healthy	X		
P301L (I)	59	Female	FTDP-17T	✓	Wb, IH	Alberici *et al.* (2004)
P301L(II)	36	Female	FTDP-17T	✓		Previously undescribed case, mutation confirmed.
N279K(I)^a^	44	Female	FTDP-17T	X		O'Dowd *et al.* (2012)
N279K(II) ND32854	43	Male	FTDP-17T	✓		NINDS Coriell Biorepository^b^
N279K (III) ND40076	44	Female	FTDP-17T	✓		NINDS Coriell Biorepository^b^
*N279K*	*45*	*Male*	*FTDP-17T*	*–*	*Wb*	Delisle *et al.* (1999)
*P301L*	*55*	*Female*	*FTDP-17T*	*–*	*IH*	Mirra *et al.* (1999)
*P301L*	*66*	*Male*	*FTDP-17T*	*–*	*IHr*	Spillantini *et al.* (1998*a*)
*P301L*	*53*	*Male*	*FTDP-17T*	*–*	*IHr*	Spillantini *et al.* (1998*a*)
*Control*	*60*	*Female*	*–*	*–*	*IH*	
*Control*	*66*	*Male*	*–*	*–*	*IH*	

Subjects providing only brain tissue are shown in italics. IH = immunohistochemistry; IHr = immunohistochemistry where existing stained sections were re-examined; Wb = western blot.

^a^RT-PCR to detect 3R and 4R tau isoform expression was performed using RNA from IPSCs at Day 1 of differentiation also in these lines. Only 3R mRNA was present in the control case while the N279K case, similarly to the other patients with the same mutation, showed both 3R and 4R tau mRNA at this time point confirming earlier expression of 4R tau. All cases used for IPSC derivation had a MAPT H1H1 haplotype.

^b^Fibroblasts obtained from NINDS Coriell Biorepository (https://www.coriell.org/).

Culture of all hIPSC lines was carried out on irradiated mouse embryonic fibroblasts (MEFs) according to standard protocols (Chambers *et al.*, 2009). Cells were maintained in knockout serum replacement (KSR) medium (all components were from Invitrogen unless otherwise stated): Dulbecco’s modified Eagle’s medium/F12 containing 20% knockout serum replacement, fibroblast growth factor 2 (FGF2) (4 ng/ml) (PreproTech), 1 mM L-glutamine, 1 mM non-essential amino acids, 1 mM 2-mercaptoethanol, penicillin (50 U/ml), and streptomycin (50 µg/ml). Colonies were passaged once per week using a collagenase/dispase enzyme mix (Vallier *et al.*, 2009). Several IPSC lines were generated from each subject and their pluripotency was validated by expression of Oct4, Tra1-60 and SSEA4 (Supplementary Fig. 1).

### Karyotyping of IPSC lines

Karyotyping was performed by the Cytogenetics Laboratory at Addenbrooke’s Hospital (Cambridge, UK) (Supplementary Fig. 1). Briefly 0.1 µg/ml colcemid solution (Sigma) was added to each culture for 2 h in growth medium. Pluripotent stem cell colonies were then dissociated into single cells with Accutase (StemPro®Accutase®, Life Technologies) and plated on 0.1% gelatine-coated dishes for 1 h at 37°C, in an incubator with 5% CO_2_. The IPSC suspension was then collected into tubes and following centrifugation at 1200 rpm for 7 min pellets were resuspended into 5 ml of a 55 mM KCl hypotonic solution, centrifuged again at 1200 rpm for 7 min and fixed with 3:1 methanol:acetic acid before delivery to the Cytogenetics Laboratory for processing.

### Neuronal differentiation of human IPSCs

**Figure 2:**
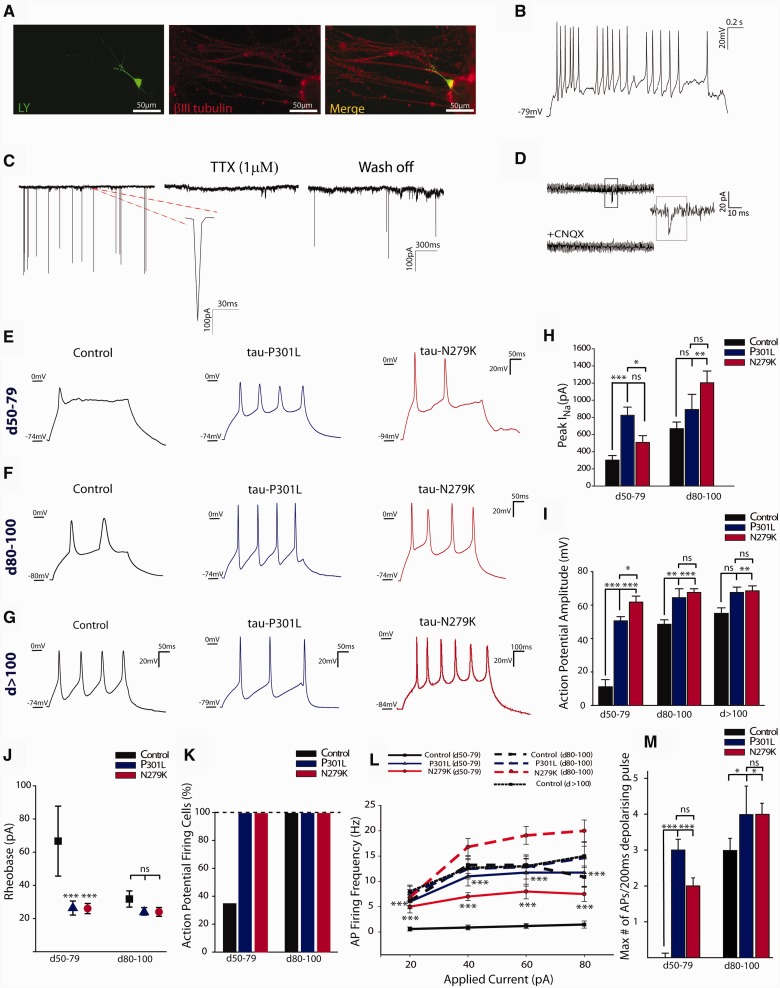
**Electrophysiological activity of control, P301L and N279K, IPSC-derived neurons.** (**A**) Lucifer yellow (LY) filled patch-clamped neuron identified post-recording by labelling for βIII-tubulin (control neuron), (**B**) spontaneous action potentials (APs) (control neuron), (**C**) large synaptic inputs reversibly blocked by tetrodotoxin (TTX, 1 µM) (P301L neuron) and (**D**) spontaneous miniature EPSCs blocked by the AMPA-receptor antagonist CNQX (20 µM) (N279K neuron), were detected in all cell lines from Day 80. Features shown in **B**, **C** and **D** are representative of control and *MAPT* mutant neurons. (**E–G**) Response to depolarizing current injection (40 pA) in (**E**) control, P301L- and N279K-derived neurons at Days 50–79, in (**F**) at Days 80–100 and in (**G**) at >Day 100. (**H**) Peak I_Na_ from neurons at Days 50–100. (**I**) Amplitude of evoked action potentials at different stages of neuronal differentiation. (**J**) Minimum current needed to evoke a single action potential (Rheobase) at different times of neuronal differentiation. Control neurons require significantly more current than *MAPT* neurons only at Days 50–79. (**K**) Percentage of cells firing action potentials at Days 50–100 showing a reduced number of control neurons firing at Days 50–79 compared to *MAPT* mutant neurons. (**L**) Average action potential firing frequency (Hz) in response to a depolarizing current pulse (20 pA to 80 pA, steps of 200 ms). (**M**) Maximum number of evoked action potentials showing a significant difference between control and *MAPT* mutant neurons at Days 50–79. Data are represented as mean ± SEM. Statistical analysis was performed using two way-ANOVA and Holm-Sidak *post hoc*. *P < 0.05, **P < 0.01, ***P < 0.001, ns = non significant.

Differentiation of human IPSCs into cortical neurons was carried out using a protocol adapted from Shi *et al.* (2012) with small modifications. Colonies were dissociated with Accutase® and plated as single cells on 24-well plates coated with CELLstart^TM^ substrate (Life Technologies) in mouse embryonic fibroblast-conditioned medium with 10 ng/ml FGF2 (Peprotech) and Rock inhibitor Y-27632 (Tocris). Once cells reached 90% confluence, medium was replaced with N2/B27 medium supplemented with 500 ng/ml mouse Noggin-CF chimera (R&D Systems) and 10 mM SB431542 (Tocris) to initiate neural induction (Day 0). On Day 12, neuroepithelial cells were dissociated with dispase (Sigma) and seeded on laminin-coated 6-well plates in N2/B7 medium. The resulting monolayer of neural precursor cells, forming tube-like rosette structures, was passaged up to three times for expansion. To enhance neuronal maturation, on Day 20, neural precursor cells were exposed to 10 µM Notch inhibitor (DAPT, Sigma Aldrich) for 96 h. Neurons were then maintained up to 200 days with a fresh medium change every other day. βIII-tubulin staining showed that more than 90% of cells in the cultures were neurons in both control and *MAPT* mutant IPSC lines. Neuronal identity was further confirmed by staining with neuronal markers, electrophysiology and RNA-seq (Figs 1 and 2 and Supplementary Fig. 2).

### Reverse transcriptase PCR and quantitative PCR

Total RNA was extracted from IPSC lines at different time points during neuronal differentiation using the RNeasy® mini kit (Qiagen) according to the manufacturer’s protocol and used for both semiquantitative and quantitative PCR. Three repeat (3R) and 4R tau mRNA expression were detected by semi-quantitative RT-PCR using the One step RT-PCR kit (Qiagen) with the following primers: forward 5′-AAGTCGCCGTCTTCCGCCAAG-3′; reverse 5′-GTCCAGGGACCCAATCTTCGA-3′. RT-PCR products were detected on a 1.5% agarose gel: 4R and 3R tau RT-PCR products were 381 and 288 bp, respectively.

For quantitative PCR, the RNA concentration was determined using a NanoDrop and 1 µg RNA was reverse transcribed to cDNA using SuperScript® First-Strand cDNA Synthesis for quantitative RT-PCR (Life Technologies). Quantitative PCR was performed with 2 × TaqMan® Fast Universal PCR Master Mix, no AmpErase UNG (Life Technologies). For 3R mRNA detection, the FAM ABI probe (ID Hs00902192_m1) that binds to the exon 9–exon 11 junction of *MAPT* was used. For 4R mRNA detection, the FAM ABI probe (ID Hs00902312_m1) that binds to the exon 9–exon 10 junction was used. *MAPT* mRNA values were normalized to those of human *GADPH* VIC TaqMan® probe (Applied Biosystems) simultaneously amplified in each sample. Triplicates were performed for each sample.

### Immunocytochemistry

The following primary antibodies were used: anti-3R tau (clone RD3, Upstate; 1:500), anti-4R tau (clone RD4, Upstate; 1:500), phosphorylation-dependent anti-tau antibodies AT8 (Innogenetics, 1:1000) and AT100 (Innogenetics, 1:1000), anti-βIII-tubulin (Millipore, 1:1000, or Covance, 1:1000), anti-alpha-synuclein antibody syn1 (BD Transduction, 1:1000), anti-β-amyloid precursor protein (Zymogen, 1:1000), anti-Oct-4 antibody (Abcam, 1:1000), anti-Tra1-60 antibody (Abcam, 1:500), anti-SSEA4 antibody (Abcam, 1:500), anti-FUS antibody (Bethyl Laboratories, 1:500) and anti-TDP-43 antibody (Protein Tech Inc., 1:1000). Immunocytochemistry was performed as previously described (Iovino *et al.*, 2010). Briefly, cells were fixed in 4% paraformaldehyde for 15 min at room temperature, washed in PBS, incubated in 5% goat serum in PBS/0.01% Tween 20 for 1 h and then incubated overnight at 4°C with primary antibodies. Staining was revealed by incubation with fluorescent-conjugated secondary antibodies (Alexa, Invitrogen Molecular Probes, 1:1000) for 1 h at room temperature. For 3R, 4R tau, AT8 and AT100 immunofluorescence, cells were incubated with biotinylated secondary antibodies (Vector Laboratories, 1:250) for 2 h at room temperature and 1 h with fluorescent streptavidin (Alexa, Invitrogen, 1:500). Double-labelling immunofluorescence was performed by sequential incubation with the appropriate primary antibodies. Cell nuclei were visualized with Hoescht dye (Sigma-Aldrich, 1:5000). Specificity of staining, when necessary, was determined by pre-adsorption of the primary antibody.

### Immunohistochemistry of brain tissue sections

Following fixation in 10% buffered formalin, half of the P301L *MAPT* mutant patient brain was sliced in the coronal plane and frontal cortex used for histological examination. The other half of the brain was sliced in the coronal plane, flash frozen and stored at −80°C. Paraffin-embedded sections (7 μm) from frontal cortex were deparaffinized, rehydrated and endogenous peroxidase activity blocked with methanol/0.3% H_2_O_2_. Immunohistochemistry was performed as previously reported (Spillantini *et al.*, 1998*a*). Briefly sections were incubated overnight at 4°C with primary antibody, washed, and incubated at room temperature for 2 h with biotinylated secondary antibody (1:250, Vector Laboratories), before incubation for 1 h with Avidin-DH (1:100, Vectastain Elite Kit, Vector laboratories). Staining was developed using 3,3 diaminobenzidine/H_2_O_2_ and sections counterstained with cresyl violet before mounting in DPX (Sigma-Aldrich). For immunofluorescence, following incubation with biotinylated secondary antibody, sections were incubated with fluorescent streptavidin (Alexa, Invitrogen). Paraffin sections from cerebral cortex of two control subjects (Table 1) were obtained from the Cambridge Brain Bank and processed as indicated above.

### Soluble tau extraction and immunoblotting

Soluble tau was extracted at different time points of neuronal differentiation from 5 × 10^5^ cells per line using 2.5% perchloric acid (Sigma-Aldrich) as previously described (Iovino *et al.*, 2010). Tau extract was dephosphorylated with 0.3 units/µl of alkaline phosphatase from *Escherichia coli* (Sigma-Aldrich) in 50 mM Tris-HCl and 5 mM MgCl_2_ for 3–4 h at 65°C. Proteins were separated by 10% SDS-PAGE and transferred onto Immobilon-P membranes (Millipore). Membranes were then probed with polyclonal anti-human tau antiserum (Dako, 1:1000) followed by peroxidase-conjugated secondary antibody (Dako, 1:10 000). Soluble tau was also extracted as described above from the cerebral cortex of one of the patients with the P301L mutation (Alberici *et al.*, 2004) and cortex from a N279K patient whose tissue was obtained from the Indiana University Alzheimer Centre Tissue Bank (Table 1). Experiments were repeated at least three times using different cell extracts.

### MAP2 extraction and immunoblotting

For MAP2 protein immunoblotting, extracts were produced from control and *MAPT* mutant cell lines using RIPA Buffer (Sigma-Aldrich) supplemented with complete protease inhibitor cocktail (Roche). Extracts were separated by 4–12% SDS-PAGE and immunoblotted using anti-MAP2 antibody (Cell Signalling, 1:500). As loading control, blots were reprobed with an anti-actin antibody (Abcam, 1:1000).

### Mitochondrial axonal transport

Human IPSC-derived neurons from control, P301L and N279K lines at 65 days of differentiation (d65) were labelled with 10 nM MitoTracker Orange (Invitrogen) for 5 min at 37°C. Cells were then washed three times and fresh culture medium was added. Mitochondrial dynamics along the neuronal processes were recorded with a 63× oil immersion objective using with a Hamamatsu EM CCD C9100 camera (Leica AF7000). The environment was controlled with a heated stage set at 37°C and 5% CO_2_. Time-lapse images of mitochondria were acquired every 6 s for 5 min (300 s; 50 frames in total). For each IPSC line, the proportion of stationary, anterograde and retrograde moving mitochondria were calculated as described previously (Mellone *et al.*, 2013). Mitochondria for each cell line were analysed in three different experiments, the total number of mitochondria analysed for each line was: controls *n* = 270; N279K *n* = 203; P301L *n* = 371. Results were evaluated by one-way ANOVA followed by Bonferroni *post hoc* correction after checking for Gaussian normality by D'Agostino and Pearson omnibus test (Graph Pad Prism).

### Electrophysiological recordings

Whole-cell current- and voltage-clamp recordings (Multi-clamp 700B, Axon Instruments) were performed as previously described (Lundgaard *et al.*, 2013) but with K^+^ glyconate as internal solution for the electrodes. Cultures were superfused with HEPES-buffered external solution bubbled with medical oxygen consisting of 144 mM NaCl, 2.5 mM KCl, 10 mM HEPES, 1 mM NaH_2_PO_4_, 2.5 mM CaCl_2_, 10 mM glucose, pH 7.35 (adjusted with NaOH), with fresh addition of 2 mM MgCl_2_ prior to the recordings. In some experiments, 1 μM tetrodotoxin (TTX) was applied to block voltage-gated sodium channels and neuronal activity. Recordings were corrected for −14 mV junction potential and data were analysed using Clampfit 10.2 and Sigma Plot 11. Following completion of the recordings, coverslips were fixed with 4% paraformaldehyde for 15 min at room temperature and further immunostained for either βIII-tubulin (Sigma, T8660, 1:1000) or Neurofilament (Sigma, N2912, 1:1000). Two-way ANOVA and Holm-Sidak *post hoc* tests were used for statistical analysis (Graph Pad Prism).

### RNA-seq data analysis

RNA from six IPSC lines from two healthy control subjects, two related patients carrying the P301L mutation in the *MAPT* gene and two patients carrying the *MAPT* N279K mutation, was extracted as described for RT-PCR at Day 0. Additionally, RNA was extracted from five of the IPSCs (two control, two P301L and one N279K) at Day 65. Paired-end barcoded Illumina RNA libraries were prepared using the standard Illumina protocol and sequenced on the Illumina HiSeq 2000. In addition, RNA-seq expression data were downloaded from human normal brain temporal lobe mRNA and normal total brain RNA (Twine *et al.*, 2011) from the European Nucleotide Archive (run accession numbers SRR085471 and SRR085725, respectively). As a further control, RNA-seq data from the H1 embryonic human stem cell line published by the ENCODE consortium were downloaded (Kwasnieski *et al.*, 2014).

The sequencing data for all of the IPSC samples, ENCODE cell lines and brain samples were aligned using TopHat v2.0.4 (Kim *et al.*, 2013) with default parameters to the GRCh37 version of human reference genome using the annotation from ENSEMBL (release 69) as a guide. A set of custom scripts and samtools (v 0.1.18) were used to filter reads to obtain reads that mapped uniquely, allowing up to three mismatches, minimum mapping quality of 10, minimum and maximum insert size of 150 and 1 000 000, respectively. Subsequently reads or fragments that aligned to each gene based on ENSEMBL annotation were counted using a customized script. Using the counts obtained, the number of fragments per kilobase per million mapped reads (FPKM) for all of the protein coding genes in ENSEMBL (release 69) annotation of the GRCh37 genome was calculated. Pearson correlation coefficients in FPKM values across all samples, were computed as well as the average correlations using a Fisher Z transform.

### Genetic analysis of H1 and H2 MAPT haplotype

Genomic DNA was extracted from each IPSC line with QIAamp DNA mini kit (Qiagen) according to the manufacturer’s protocol. Genetic analysis of H1 and H2 haplotypes SNP genotyping for rs9468, tagging the *MAPT* H1 versus H2 haplotype, was performed using the TaqMan® allelic discrimination assay on an HT7900 sequence detection system (Applied Biosystem), according to the manufacturer’s instructions.

## Results

### Expression of tau isoforms in human IPSC-derived neurons

In control and P301L cells, 3R tau mRNA was present from Day 1 of neuronal differentiation (d1) while 4R tau mRNA appeared at Day 30 (d30, Fig. 1B). N279K neurons instead showed both 3R and 4R tau mRNA from Day 1 (Fig. 1B). A similar pattern in the expression of 4R tau mRNA over time was also detected by quantitative PCR (Supplementary Fig. 3).

Tau protein detected by immunocytochemistry was present in all neurons at Day 30, 3R tau being found in cell bodies and neuronal processes (Fig. 1D). However, in control and P301L neurons, 4R tau was only present in cell bodies and some processes around Day 55, whereas in N279K neurons, 4R tau was already detected at Day 30, at the same time as 3R tau (Fig. 1D), with a distribution similar to that in control and P301L neurons at Day 55. Hence, there was a much earlier expression of 4R tau in N279K neurons compared to that in control and P301L neurons.

The tau protein isoform expression was further investigated by immunoblotting of IPSC-derived neuron extracts at Days 0, 55 and 150, and compared to patients’ brain extracts. At Day 0 a weak band corresponding to 0N3R tau protein could be inconsistently seen (data not shown). At Day 55, in control and P301L neurons the embryonic 0N3R tau isoform was strongly expressed, in some experiments a band corresponding to 0N4R tau could be clearly seen (Supplementary Fig. 4), this would be in agreement with the immunocytochemistry data indicating 4R tau expression at Day 55. It is possible that in blots where it is not visible the abundant 3R tau band masks the weaker 4R tau band. In N279K neurons bands corresponding to 3R and 4R tau isoforms were clearly visible at Day 55 (Fig. 1C). The remaining tau isoforms were detected around Day 150 in all lines. Notably, at Day 150 the tau isoform profiles in P301L and N279K neurons were similar to those found in extracts of brain tissue obtained from patients with the respective mutations (Fig. 1C and Table 1); the tau profile in control neurons was similar to that of P301L brain extract. In both control and P301L neurons, the shortest 0N3R tau isoform was the predominant isoform at Day 150, similar to differentiating human embryonic stem cells (Iovino *et al.*, 2010) probably due to recruitment of newly differentiating neurons only expressing 0N3R tau protein. A schematic diagram of the time course of tau mRNA and protein expression is shown in Supplementary Fig. 4. The shortest 0N3R and 1N3R tau isoforms were relatively less abundant in the N279K mutant neurons compared to the control and P301L neurons, suggesting an effect of the mutation on the splicing of 3R tau isoforms. The N279K mutation might act on a proposed purine-rich exonic splice enhancer that reduces the relative expression of some 3R tau isoforms (D’Souza *et al.*, 1999). In fact, a reduction in the 1N3R tau isoform is present in the N279K patient brain extracts as also previously shown (Clark *et al.*, 1998). Together these results support the existence of an imbalance in 3R:4R tau isoform expression and show the novel finding that 4R tau expression occurs earlier during neuronal development in N279K neurons.

### Earlier maturation of human IPSC-derived neurons from subjects with *MAPT* mutations

**Figure 3:**
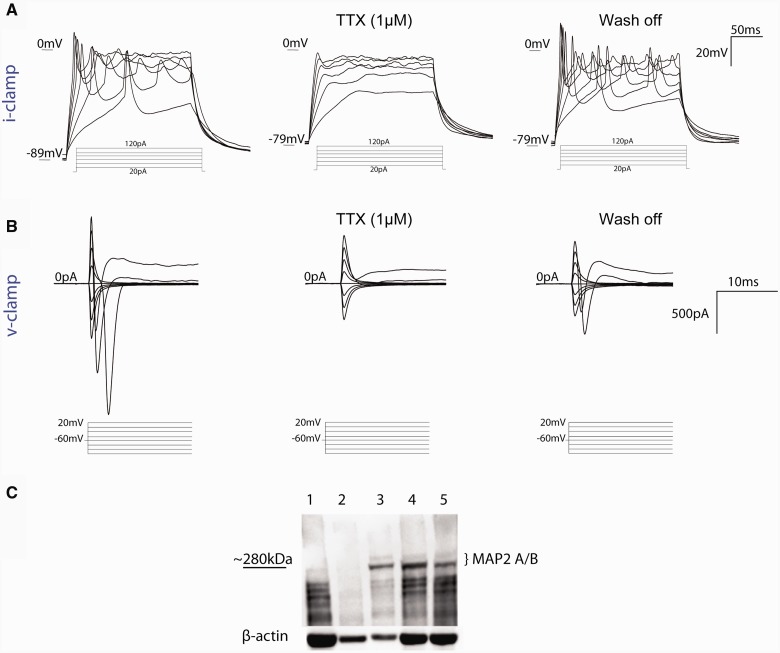
**IPSC-derived neurons have TTX sensitive I_Na__v_ and action potentials.** (**A**) Responses to current injections (current-step range from 20–120 pA: 200 ms) in IPSC-derived neurons, are reversibly blocked by the voltage-gated Na^+ ^channel blocker TTX (1 µM) (Days 80–100, control neuron). (**B**) TTX (1 µM) sensitive I_Na _(20 mV, voltage range −120 mV to +20 mV; 200 ms) of IPSC-derived neuron held at −70 mV (Days 80–100, control neuron). (**C**) Immunoblotting of extracts of IPSC-derived neurons with anti-MAP2 antibody control neurons Day 35 (lane 1), Day 63 (lane 2); P301L neurons Day 65 (lane 3); N279K neurons Day 70 (lane 4), Day 63 (lane 5). High molecular weight post-natal MAP2 A and B isoforms are clearly visible in *MAPT* mutant IPSC-derived neurons. β-actin is used as loading control.

To examine the functional differences between the IPSC-derived neuron lines, we used whole-cell patch-clamp to record the neurons at different time points during neuronal differentiation (Days 50–79, Days 80–100 and >Day 100; Fig. 2). The interval between Days 50–79 of neuronal differentiation was selected as first time point for electrophysiology because at this time cell lines expressed both 3R and 4R tau, as shown by PCR, immunocytochemistry and immunoblotting. Differentiated cells matured into functional glutamatergic neurons and generated a neuronal network *in vitro*, as detected by spontaneous repetitive action potentials (Fig. 2B), large synaptic inputs reversibly blocked by 1 μM TTX (Figs 2C, 3A and B), and spontaneous miniature EPSCs blocked by the AMPA receptor antagonist CNQX (20 μM; Fig. 2D). Membrane capacitance, potential, and resistance were similar between all lines. However, there were stark differences between lines in the time course of neuronal maturation. Control neurons phenocopied developmental neuronal maturation (Picken Bahrey and Moody, 2003); at early time points 36% (5/14) of neurons generated a single immature action potential (Fig. 2E and K) in response to a large depolarizing current (Fig. 2J), mostly due to low expression of voltage-gated sodium channels (Na_v_) (Fig. 2H). However, as the Na_v_ current increased (Days 50–79 versus Days 80–100; *P* = 0.007, Fig. 2H), the minimum injected current needed to elicit an action potential decreased (rheobase; 66.7 ± 21.1 pA; *P* < 0.001, Fig. 2J); this was followed by detection of (TTX-sensitive) regenerative action potentials (Figs 2C and 3A), which increased in frequency with larger current pulses and had mature action potential amplitude (Fig. 2I and L). On the other hand, injecting depolarizing currents into P301L and N279K neurons evoked regenerative action potentials in 100% of cells at all time points (Fig. 2E–G and K), which increased in frequency with larger current injections (Fig. 2L). Moreover, the neurons with *MAPT* mutations fired more action potentials per injected 200 ms current pulse (Fig. 2M), with taller and more mature action potential amplitude compared to control neurons (Fig. 2I and E–G). Thus, we conclude that neurons with P301L and N279K *MAPT* mutations mature faster, as also supported by the higher expression of postnatal MAP2A/B isoform in *MAPT* mutant neurons (Fig. 3C), that were also more excitable.

### Characterization of pathological features in human IPSC-derived neurons with *MAPT* mutations

**Figure 4:**
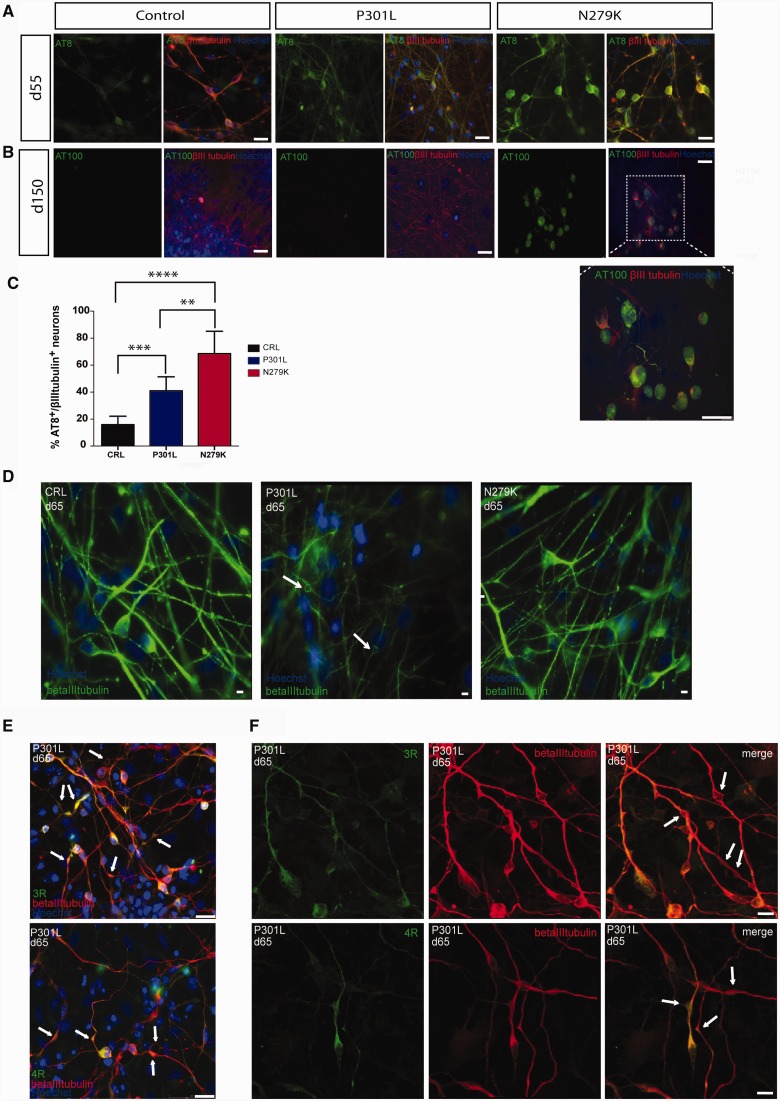
**Tau hyperphosphorylation and morphological characterization of IPSC-derived neurons.** (**A**) Immunocytochemistry of control, P301L and N279K IPSC-derived neurons at Day 55 using the phosphorylation-dependent anti-tau antibody AT8 (green), βIII-tubulin (red) and Hoechst (blue). Staining for AT8 is present in several P301L and N279K neurons where it often consists of ring-like structures. (**B**) Immunocytochemistry for phosphorylation-dependent anti-tau antibody AT100 (green), βIII-tubulin (red) and Hoechst (blue) in control, P301L and N279K neurons at Day 150. Some N279K neurons show AT100 staining in cell bodies. The image magnification in the insert shows accumulation of tau protein stained by AT100 in neurites and in cell bodies where sometimes the staining has a dotted appearance. (**C**) Statistical analysis of the AT8-positive neurons within the βIII-tubulin positive cells shows a significant increase in tau hyperphosphorylation in P301L and N279K neurons compared to controls (CRL). Cell count was performed using ImageJ and statistical analysis was evaluated with Student’s *t*-test. **P < 0.006; ***P < 0.007; ****P < 0.001. (**D**) Staining with βIII-tubulin (green) of control, P301L and N279K neurons at Day 55 shows only in P301L neurons abnormal processes with varicosity-like structures indicated by arrows. (**E**) Immunocytochemistry for 3R (green, *top panel*), 4R (green, *bottom panel*) tau isoforms, βIII-tubulin (red) and Hoechst (blue) shows that the varicosity-like structures are positive for 3R and 4R tau isoforms. (**F**) Confocal images of P301L neurons with 3R (green, *top panels*) 4R (green, *bottom panels*) tau and βIII-tubulin (red) at Day 65 confirm the presence of the varicosity-like structures indicated by arrows. Scale bars = 25 µm.

Abnormal tau hyperphosphorylation is a hallmark of tauopathies (Goedert *et al.*, 1992; Spillantini and Goedert, 2013). At Day 55, 41% of P301L and 68% of N279K neurons were positive for the phosphorylation-dependent anti-tau antibody AT8, a significant increase compared to the 15.8% of control neurons (*P* < 0.007 and *P* < 0.001, respectively) (Fig. 4A and C). Moreover, phospho-tau in cell bodies often formed ring-like structures similar to those in FTDP-17T brains (Spillantini *et al.*, 1998*a*) and mice transgenic for human P301S tau (Hampton *et al.*, 2010). The AT100 antibody (Zheng-Fischofer *et al.*, 1998; Yoshida *et al.*, 2006), a late marker of tau hyperphosphorylation and indicator of filamentous tau aggregates (Allen *et al.*, 2002), stained some neuronal cell bodies, sometimes showing dot-like structures only in the N279K lines at Day 150 (Fig. 4B).

**Figure 5:**
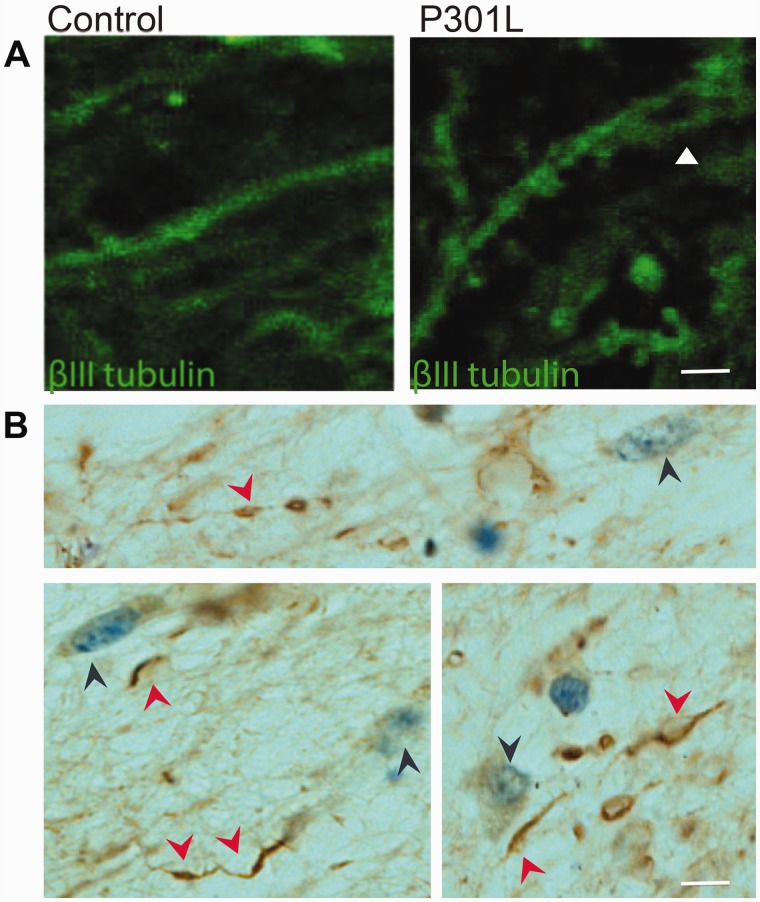
**Immunohistochemistry of brain sections from the cortex of the patient with the P301L mutation and a control subject.** (**A**) Confocal microscope image of control and P301L human brain frontal cortex sections stained with anti-βIII-tubulin antibody. The arrow points to a varicosity-like structure. No similar structures were found in the human control brain. (**B**) Examples of varicosity-like structures stained by phosphorylation-dependent anti-tau antibody AT8, in the frontal cortex of the P301L patient from whom IPSCs were generated. Red arrows point to varicosity-like structures. Blue arrows point to cell bodies where nuclei are stained by cresyl violet. Scale bars: **A** = 17 μm; **B** = 68 µm.

Morphological differences were evident between lines, although both N279K and P301L neurons had some thicker processes than control neurons. P301L neuronal processes were more contorted with varicosity-like structures appearing after Day 55, stained by βIII-tubulin, some containing 3R and 4R tau (Fig. 4D and F). The varicosity-like structures were present only in neurons from the two patients with the P301L mutation; they were not present in neurons from the control subjects or patients with the N279K mutation. We detected similar varicosity-like structures in the brain of the patient from whom the P301L neurons were derived (Fig. 5) and other patients with the same mutation (Spillantini *et al.*, 1998*a*; Mirra *et al.*, 1999).

**Figure 6:**
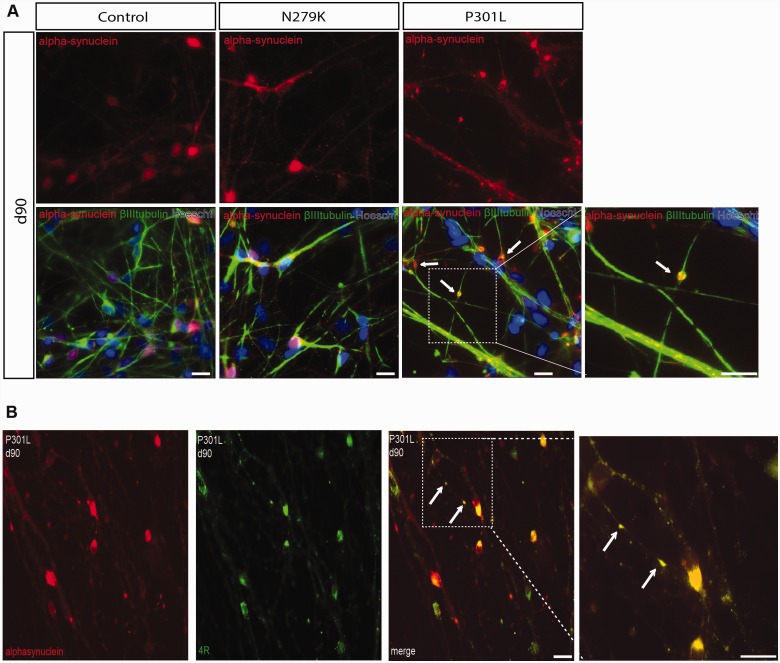
**Alpha-synuclein staining in IPSC-derived neurons.** (**A**) Immunostaining for alpha-synuclein (red), βIII-tubulin (green) and Hoechst (blue) in control, P301L and N279K IPSC-derived neurons at Day 90 shows alpha-synuclein in cell bodies in all neurons, however, in P301L the protein is also present in the varicosity-like structures as indicated by arrows. (**B**) Immunostaining for alpha-synuclein (red) and 4R tau (green) in P301L neurons shows co-localization of 4R tau and alpha-synuclein in the varicosity-like structures. Examples of co-localization are indicated by arrows in the enlarged area in the *inset*. Scale bars = 25 µm.

Alpha-synuclein aggregates in Parkinson’s disease (Spillantini *et al.*, 1998*b*) and it has been reported to interact with tau (Giasson *et al.*, 2003; Goris *et al.*, 2007; Satake *et al.*, 2009; Simón-Sánchez *et al.*, 2009). We therefore stained neurons for alpha-synuclein at Day 90 when neurons were more mature. Positive staining was detected in cell bodies of control and N279K neurons, while in P301L neurons, staining was also found in the varicosity-like structures (Fig. 6A) where it co-localized with 4R tau (Fig. 6B).

**Figure 7:**
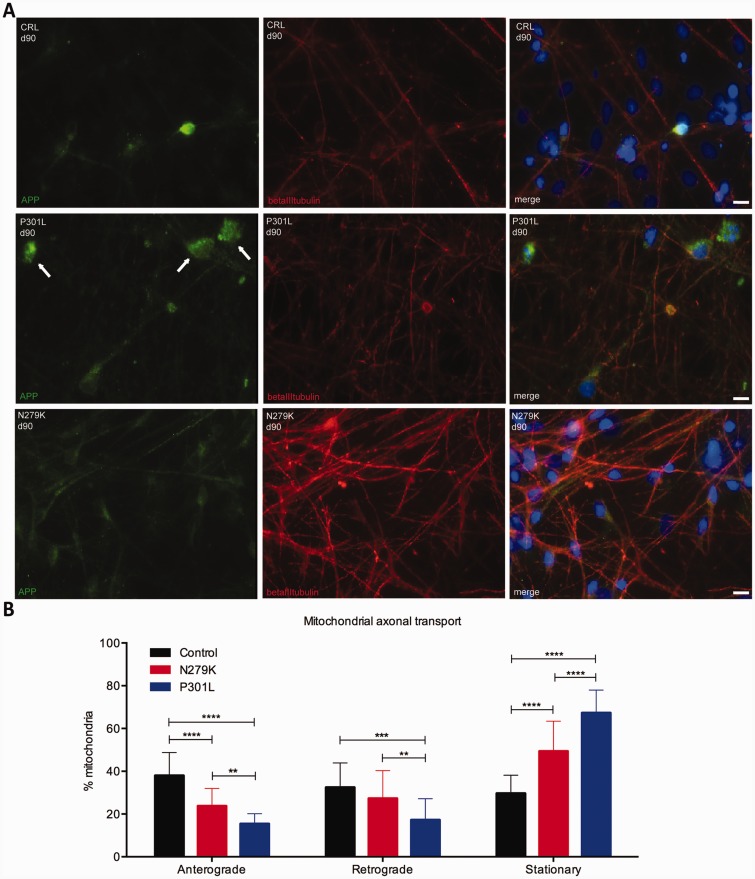
**APP expression and mitochondrial axonal transport in IPSC-derived neurons.** (**A**) Immunocytochemistry of amyloid precursor protein (APP, green), βIII-tubulin (red) and Hoescht (blue) in control, P301L and N279K IPSC-derived neurons at Day 90 (d90) shows that amyloid precursor protein accumulates in some cell bodies of P301L neurons where in some Hoescht stained a pyknotic nucleus. Arrows in the P301L neuron panel point to amyloid precursor protein stained cell bodies. Scale bar = 25 μm. (**B**) Proportion of mitochondria moving anterograde, retrograde and stationary in control, N279K and P301L neurons at Day 65. The number of anterograde moving mitochondria is reduced in N279K neurons (23% ± 2.729) and even further reduced in P301L neurons (15.30% ± 1.369) compared to control neurons (35.49% ± 4.78). Retrograde moving mitochondria are significantly reduced in P301L (16.76% ± 2.045) but not in N279K (24.09% ± 2.969 *P* = 0.5408) neurons compared to controls (24.08% ± 2.823). The percentage of stationary mitochondria is significantly increased in both N279K and P301L neurons compared to controls. For every imaged axon the number of anterograde and retrograde moving and stationary mitochondria is expressed as a percentage where the total number of neurons examined per each line (100%) was control *n* = 270; N279K *n* = 203; P301L *n* = 371. Data are represented as mean ± SD of three independent experiments. Statistical analysis was performed by one-way ANOVA followed by Bonferroni *post hoc* correction after checking for Gaussian normality by D'Agostino and Pearson omnibus test. *P < 0.05, **P < 0.01, ***P < 0.002, ****P < 0.001.

Immunohistochemistry for the amyloid precursor protein (Nicolas and Hassam, 2014) performed at Day 90 showed its accumulation in some cell bodies only in P301L neurons (Fig. 7A) some of which had pyknotic nuclei. No difference in TDP43 and FUS (Da Cruz and Cleveland, 2011) staining was found between mutant cells and controls (Supplementary Fig. 5).

### Mitochondrial axonal transport is altered in *MAPT* mutant IPSC-derived neurons

We investigated whether axonal transport is altered in P301L and N279K neurons compared to controls by imaging axonal mitochondrial movement at Day 65. The percentage of anterogradely moving mitochondria was significantly reduced in *MAPT* mutant neurons (N279K: 23% ± 2.729 *P* = 0.03; P301L: 15.30% ± 1.369 *P* = 0.002) compared to controls (35.49% ± 4.78) (Fig. 7B). However, the retrograde movement of mitochondria was only reduced in P301L neurons (*P* = 0.01: Fig. 7B), as might be predicted from its specific effects on microtubule stability.

## Discussion

Here we show for the first time that human IPSC-derived neurons can express the tau isoforms present in human adult brain. Remarkably, neurons with P301L and N279K *MAPT* mutations mature faster and are more excitable than neurons from control subjects. Similarly, neurons from mice overexpressing human P301L tau (Crimins *et al.*, 2012) and familial frontotemporal dementia patients (Kuhn *et al.*, 2004) have shown increased excitability. As neuronal activity influences tau release (Pooler *et al.*, 2013), it is therefore possible that the abnormal earlier maturation and increased excitability of the neurons from patients with *MAPT* mutations contribute to their faster ageing, with tau hyperphosphorylation and accumulation occurring through altered tau release. However, the P301L and control lines do not significantly differ in the expression of 4R tau, therefore, the reason for the earlier P301L neuron maturation is not clear. Future work will aim to determine whether it is only the reduced microtubule-binding due to the P301L mutation that is responsible for the earlier neuronal maturation or instead if there are other effects of the mutation that have not yet been detected or predicted.

Besides the common feature of faster maturation, neurons from FTDP-17T patients present a mutation-specific phenotype in accordance with their predicted effects on destabilizing microtubules (P301L) or tau mRNA splicing (N279K). P301L neurons are specifically characterized by abnormal varicosity-like structures, sometimes containing alpha-synuclein, amyloid precursor protein accumulation and deficits in retrograde axonal transport. Indeed, the absence of a retrograde axonal transport deficit in the N279K neurons could partially account for the finding of alpha-synuclein and amyloid precursor protein accumulation specifically in neurons with the P301L mutation. Interestingly, the varicosity-like structures in the P301L mutant neurons were also found in the brain of one of the patients from whom the P301L neurons were derived (Fig. 5) and other patients with the same mutation (Spillantini *et al.*, 1998*a*; Mirra *et al.*, 1999). These varicosity-like structures look similar to ‘neuronal parcels’ (Hollenbeck and Bray, 1987) and could represent branch points where alpha-synuclein has been reported to accumulate (Kanazawa *et al.*, 2012). If this localized interaction between 4R tau and alpha-synuclein was deleterious, it could explain why the H1 *MAPT* haplotype, associated with increased 4R tau (Caffrey *et al.*, 2006; Myers *et al.*, 2007), is a risk factor for Parkinson’s disease (Satake *et al.*, 2009; Simón-Sánchez *et al.*, 2009). All the controls and *MAPT* mutant patients used in this study carried a *MAPT* H1H1 haplotype.

The N279K neurons instead are characterized by premature developmental expression of 4R tau, changes in 3R:4R isoform ratio and AT100 staining of hyperphosphorylated tau, suggestive of tau aggregation possibly related to early 4R tau expression. The novel finding of abnormal developmental expression of 4R tau, together with the imbalance of 3R:4R tau, could critically contribute to FTDP-17T, adding a developmental twist to this complex disorder. Future studies will need to investigate whether other *MAPT* splicing mutations are associated with earlier developmental expression of 4R tau.

Our finding that the six adult tau isoforms can be expressed in human IPSC-derived neurons paves the way towards investigating their specific functions and contribution to dysfunction. Moreover, it is now feasible to co-culture IPSC-derived neurons with glial cells to clarify whether and how astrocytes, microglia and oligodendrocytes contribute to development of tau pathology. Our demonstration that *MAPT* mutations cause common and specific phenotypes will improve the understanding of disease mechanisms prompting the search for new therapies for tauopathies.

## Supplementary Material

Supplementary Fig. 1

## Abbreviations

FTDP-17T: frontotemporal dementia and parkinsonism linked to chromosome 17
IPSC: induced pluripotent stem cell
TTX: tetrodotoxin
