# Supplementary material for: Early maturation and distinct tau pathology in induced pluripotent stem cell-derived neurons from patients with MAPT mutations
Source: Brain. 2015 Jul 28;138(11):3345–59. doi: 10.1093/brain/awv222 (PMC4620511; doi:10.1093/brain/awv222)
Supplement: Supplementary Fig. 1 [file suppl_data.zip › brain-2015-00254-File010.pdf]

Supplementary Methods

MRI Acquisition

Diffusion-weighted images with 42 unique gradient directions (b=1000 sec/mm^2) were collected with either seven images (Siemens) with no diffusion weighting or one image with no diffusion weighting (Phillips). For scans collected in London, dimensions were 128 pixels × 96 pixels × 65 slices per volume, with TE=84ms and TR=7600ms; for Paris, dimensions were 128 pixels × 128 pixels × 75 slices per volume, with TE=88ms and TR=13100ms and for Leiden, dimensions were 112 pixels × 112 pixels × 55 slices per volume, with TE=56ms and TR=8078ms. Voxel size for Siemens scans was 2x2x2mm^3 and for Phillips 1.96 x 1.96 x 2mm^3.

T1-weighted image volumes were acquired using a 3D MPRAGE acquisition sequence on 3.0 T Siemens (London and Paris) and Phillips (Leiden) whole body imager with the following imaging parameters: TR = 2200ms (Siemens); 7.7ms (Philips); TE=2.2ms (Seimens); 3.5ms (Philips); flip angle =10◦ (Seimens); 8◦ (Philips); FOV= 28cm (Seimens); 24cm (Philips); matrix size 256x256 (Seimens); 224x224 (Philips), 208 (Seimens); 164 (Philips) sagittal slices to cover the entire brain with a slice thickness of 1.0 mm with no gap.

Head coil information: Siemens Tim Trio 3T (London/Paris) - 12 channel head matrix with HEA (head coil element anterior)/HEP (head coil element posterior) coils selected; Philips Achieva 3T (Leiden) - 8 channel SENSE (SENSitivity Encoding) head coil.

80-region graph theory analysis

In order to ensure the exclusion of the globus pallidus and nucleus accumbens did not affect our results an 80-region graph theory and clinical correlation analysis, with the inclusion of the globus pallidus and nucleus accumbens, was performed. This was in line with the 76-region analysis outlined in the methods section of the main text (volume un-normalised, threshold 75%). Rich club regions, defined as the top 12 brain regions with the highest degree, were also identified for both the 80-region analysis combined site and at single sites to ensure the reliability of our tractography method.

For rich club regions (left superior frontal and right caudate) and global metrics showing significance correlation with emotion recognition, partial correlations were further performed across groups to investigate the association between brain networks changes and emotion

recognition in Huntington's disease, premanifest Huntington's disease and controls separately. For Huntington's disease and premanifest Huntington's disease age, sex, site, education and CAG were included as covariates. For the control group age, sex, site and education were included as covariates. Emotion recognition was chosen as scores show greater variability in controls compared to TMS therefore allowing comparison between Huntington's disease, premanifest Huntington's disease and controls.

Supplementary Results

Rich club regions were in perfect agreement across groups for both combined and single site analyses. The regions were as follows: Caudate, thalamus, superior frontal, superior parietal, precuneus and insula bilaterally.

Regional brain network measures

Similarly to the 76 region analysis a reduction in degree was seen between premanifest Huntington's disease and controls in the right caudate ($p = 0.008$) and approached significance, after correction for multiple graph metrics ($p < 0.0125$), in the left caudate ($p = 0.016$), left anterior cingulate ($p = 0.018$) and left putamen ($p = 0.018$). Manifest vs. premanifest Huntington's disease showed significant reductions in the left ($p = 0.004$) and right caudate ($p = 0.008$), left ($p = 0.0064$) and right thalamus ($p = 0.0064$), right putamen ($p = 0.0064$), right nucleus accumbens ($p = 0.004$) and right paracentral regions ($p = 0.008$). Numerous regions showed significant decreases in Huntington's disease vs. controls including cortical and basal ganglia rich club regions, as well as cingulate, motor, temporal and occipital areas (see Figure S1).

Significant increases in (graph theory) strength between premanifest Huntington's disease and controls were seen in the left ($p = 0.004$) and right caudate ($p = 0.004$). No significant differences were seen in Manifest vs. premanifest Huntington's disease. Huntington's disease vs. controls showed significant increases in the left caudate ($p = 0.002$) and decreases in the right caudate ($p = 0.002$), left ($p = 0.002$) and right putamen (p = 0l002), left nucleus accumbens ($p = 0.003$) and left postcentral regions ($p = 0.008$). Clustering coefficient showed significant decreases in premanifest Huntington's disease vs. controls in the left caudate ($p = 0.008$). In manifest Huntington's disease vs. premanifest Huntington's disease significant decreases were seen in the left ($p = 0.004$) and right putamen ($p = 0.004$).

Network segregation

Similarly to the 76-region analysis significant increases were seen in normalised clustering coefficient in Huntington's disease vs. controls ($p = 0.0001$), Huntington's disease vs. premanifest ($p = 0.001$) and premanifest vs. controls ($p = 0.0041$). Modularity showed significant increases in Huntington's disease vs. controls ($p = 0.0001$) and in premanifest Huntington's disease vs. controls ($p = 0.0018$).

Network Integration

Normalised average path length showed significant increases in Huntington's disease vs. controls ($p = 0.0001$) and manifest vs. premanifest Huntington's disease ($p = 0.0007$). Significant decreases were seen in global efficiency in Huntington's disease vs. controls ($p = 0.006$). No significant group differences were seen in small worldness.

Regional brain network clinical correlations

Clinical measures revealed correlations with the degree of rich club and non-rich club brain regions (DF = 86, for all clinical correlations). For TMS correlations were seen with the left (Rho = -0.48, $p = 2.7 \times 10^{-4}$) and right inferior parietal (Rho = -0.42, $p = 0.002$), left caudal middle frontal (Rho = -0.46, $p = 3.5 \times 10^{-4}$), left rostral middle frontal (Rho = -0.41, $p = 0.002$) and left superior frontal (Rho = -0.41, $p = 0.002$). Indirect circle tracing correlated with the left superior frontal (Rho = 0.44, $p = 0.001$), right lingual (Rho = 0.43, $p = 0.002$) and left temporal pole (Rho = 0.47, $p = 0.001$), while Negative Emotion Recognition test performance correlated with the right caudate (Rho = 0.49, $p = 2 \times 10^{-4}$) and left inferior parietal (Rho = 0.44, $p = 0.001$). No significant correlations were seen with any other regional graph metric and SWR, SDMT or Speeded Tapping mean inter-tap interval.

Whole brain network clinical correlations

The alterations in whole brain topology that are reported above showed significant correlations with cognitive and motor deficit. Network segregation: normalised clustering coefficient showed significant correlations with TMS (Rho = 0.41, $p = 1.2 \times 10^{-4}$), indirect circle tracing (Rho -0.35, $p = 0.002$) and emotion recognition (Rho = 0.44, $p = 4.1 \times 10^{-5}$), while modularity significantly correlated with TMS (Rho = 0.34, $p = 0.002$). Network integration: Correlations approaching significance were seen for normalised average path length and TMS (Rho = 0.32, $p = 0.003$).

Correlations with emotion recognition across groups

Significant correlations were seen across groups for right caudate degree and emotion recognition (See figure S2(a)). For manifest Huntington's disease significant correlations were seen for left superior frontal degree and emotion recognition (See figure S2(b)). Normalised clustering coefficient showed significant correlations with emotion recognition for manifest and premanifest HD (See figure S2(c)).

Supplementary Discussion

The 80-region analysis (with the inclusion of the globus pallidus and nucleus accumbens) revealed similar findings to the 76-region analysis, with the exception of (graph theory) strength. This is to be expected as neither of these regions have ever been defined as rich club regions (van den Heuvel and Sporns, 2011, van den Heuvel *et al.*, 2013). In the 80-region analysis premanifest Huntington's disease vs. controls show increases in (graph theory) strength in the left and right caudate while in the 76-region analysis decreases were seen in these regions, similarly increases in strength were seen in Huntington's disease vs. controls in the left caudate. These may be due increased strength of connections of the globus pallidus and nucleus accumbens to the caudate in Huntington's disease. However these results must be interpreted with caution, as parcellations for the globus pallidus and nucleus accumbens were poor and thus excluded from the main 76-region analysis.

Bogaard and colleagues (van den Bogaard *et al.*, 2011) found the nucleus accumbens and globus pallidus show grey matter atrophy in premanifest Huntington's disease. However this was found only in those premanifest subjects that were close to onset. We find reduced degree of the globus pallidus and nucleus accumbens (bilaterally) and reduced (graph theory) strength in the left nucleus acumbens in Huntington's disease vs. controls (see figure S1) however no group differences were found for graph metrics of these structures in premanifest Huntington's disease vs. controls. This may be due to grey matter atrophy of these structures occurring before loss of white matter connections or because our analysis was conducted on all premanifest subjects and not only in those close to onset.

Clinical correlations between graph theory metrics and emotion recognition across groups showed significant positive correlation with degree of right caudate and emotion recognition performance. This confirms that reduction in degree of the caudate rich club basal ganglia

region is clinically relevant in manifest and premanifest Huntington's disease. Of interest significance correlation is also seen in the control group suggesting loss of brain connections (degree) to the caudate is also of clinical importance in normal aging. Indeed impaired emotion recognition has been shown previously in an aging population (Demenescu *et al.*, 2014). Only the manifest Huntington's disease group showed significant correlation with emotion recognition and degree of the left superior frontal. This is to be expected as only Huntington's disease vs. controls show significant degree reductions in this area thus confirming loss of brain connections (degree) to cortical rich club regions are clinically important in manifest disease. For normalised clustering coefficient significant correlations are seen with emotion recognition in manifest and premanifest Huntington's disease. This is inline with significant differences in normalised clustering coefficient for both manifest and premanifest participants compared to controls and confirms that these global network changes are clinically relevant in both patient groups.

Supplementary References

Demenescu LR, Mathiak KA, Mathiak K. Age- and gender-related variations of emotion recognition in pseudowords and faces. Experimental aging research. 2014;40(2):187-207.

van den Bogaard SJ, Dumas EM, Acharya TP, Johnson H, Langbehn DR, Scahill RI, et al. Early atrophy of pallidum and accumbens nucleus in Huntington's disease. Journal of neurology. 2011;258(3):412-20.

van den Heuvel MP, Sporns O. Rich-club organization of the human connectome. The Journal of neuroscience : the official journal of the Society for Neuroscience. 2011;31(44):15775-86.

van den Heuvel MP, Sporns O, Collin G, Scheewe T, Mandl RC, Cahn W, et al. Abnormal Rich Club Organization and Functional Brain Dynamics in Schizophrenia. JAMA Psychiatry. 2013:1-10.
