# Supplementary material for: Early maturation and distinct tau pathology in induced pluripotent stem cell-derived neurons from patients with MAPT mutations
Source: Brain. 2015 Jul 28;138(11):3345–59. doi: 10.1093/brain/awv222 (PMC4620511; doi:10.1093/brain/awv222)
Supplement: Supplementary Fig. 1 [file suppl_data.zip › brain-2015-00254-File013.pdf]

Supplementary Tables

Table S1. Patient demographics

	HD	Premanifest HD	Control	Statistical test	P-value
**N**	38	50	47	-	
**Age** (SD)	49.5 (10.4)	42.2 (8.9)*	47.6 (9.0)	ANOVA	0.001
**Gender** (M/F)	13/25	26/24	15/32	Chi-square	0.09
**Education** (SD)	3.89 (1.01)	4.22 (0.84)	3.91 (1.12)	Chi-square	0.209
**Study Site** (N) (London/Leiden/Paris)	10/15/13	13/21/16	12/20/15	ANOVA	0.999
**CAG** (SD)	43.2 (2.4)	40.3 (2.1)	-	-	-
**EYO** (SD)	-	11.1 (3.9)	-	-	-
**DBS**	370.6 (10.4)	301.3 (7.4)	-	-	-

N – participant number, SD – standard deviation, M - male, F - female, CAG - CAG repeat length, EYO – Estimated years to onset, DBS - Disease burden score

*Premanifest HD shows significant age differences between controls (p = 0.006) and HD (p = 0.0005) accounting for overall significance in the model.

Table S2. Whole brain network group differences across 0%, 25%, 50%, 75%, 100% control group thresholds. A Bonferroni correction for the 5 whole brain network metrics is applied ($p < 0.01$). NS – not significant

		Control group threshold (%)	0	25	50	75	100
Normalised clustering coeffficient (a)	Volume un-normalised	Premanifest HD vs. controls	NS	NS	NS	0.0082	NS
		HD vs. Premanifest HD	0.0072	0.0041	0.0019	0.0007	0.0001
		HD vs. controls	0.0001	0.0001	0.0001	0.0001	0.0001
	Volume normalised	Premanifest HD vs. controls	0.0057	0.002	0.002	NS	NS
		HD vs. Premanifest HD	NS	0.0096	0.0057	0.0015	NS
		HD vs. controls	0.0001	0.0001	0.0001	0.0001	0.0001
Modularity (b)	Volume un-normalised	Premanifest HD vs. controls	0.0039	0.0035	0.004	0.0037	0.0084
		HD vs. Premanifest HD	NS	NS	NS	NS	NS
		HD vs. controls	0.0001	0.0001	0.0001	0.0001	0.0001
	Volume normalised	Premanifest HD vs. controls	NS	NS	NS	NS	NS
		HD vs. Premanifest HD	0.0048	0.0035	0.0075	NS	NS
		HD vs. controls	0.0001	0.0001	0.0001	0.0001	0.0001
Normalised average pathlength (c)	Volume un-normalised	Premanifest HD vs. controls	NS	NS	NS	NS	NS
		HD vs. Premanifest HD	0.0009	0.0023	0.0009	0.0032	0.0005
		HD vs. controls	0.0001	0.0002	0.0001	0.0004	0.0001
	Volume normalised	Premanifest HD vs. controls	NS	NS	NS	NS	NS
		HD vs. Premanifest HD	NS	0.0053	NS	NS	NS
		HD vs. controls	NS	0.0019	NS	NS	NS
Global efficiency (d)	Volume un-normalised	Premanifest HD vs. controls	NS	NS	NS	NS	NS
		HD vs. Premanifest HD	NS	NS	NS	NS	NS
		HD vs. controls	0.0069	0.0049	0.0053	0.006	0.0048
	Volume normalised	Premanifest HD vs. controls	NS	NS	NS	NS	NS
		HD vs. Premanifest HD	NS	NS	NS	NS	NS
		HD vs. controls	NS	NS	NS	NS	NS
Smallworldness (e)	Volume un-normalised	Premanifest HD vs. controls	NS	NS	NS	NS	NS
		HD vs. Premanifest HD	NS	NS	NS	NS	NS
		HD vs. controls	NS	NS	NS	NS	NS
	Volume normalised	Premanifest HD vs. controls	NS	NS	NS	NS	NS
		HD vs. Premanifest HD	NS	NS	NS	NS	NS
		HD vs. controls	0.0035	NS	0.0008	NS	NS

Table S3. Regional graph metrics group differences. A Bonferroni correction of $p < 0.0125$ is applied for 4 graph metrics. NS – not significant

	Graph metric	Degree	Strength	Betweenness centrality	Clustering coefficient
**a** Volume un-normalised	Premanifest HD vs.controls	See main text	Left (p=0.008) and right caudate (p=0.008)	NS	Left caudate (p=0.008)
	HD vs. Premanifest HD	See main text	NS	NS	NS
	HD vs. controls	See main text	Left (p=0.003) and right caudate (p=0.003), left (p=0.004) and right putamen (p=0.003)	NS	Right caudate (p=0.01) and left (p=0.008) and right putamen (p=0.008)
**b** Volume normalised	Premanifest HD vs. controls	Left (p=0.001) and right caudate (p=0.008)	NS	NS	NS
	HD vs. Premanifest HD	Left (p=0.008) and right caudate (p=0.013), right putamen (p=0.008), right thalamus (p=0.008) and right paracentral (p=0.008)	**Increase** in left thalamus (p=0.008) and left hippocampus (p=0.008)	NS	NS
	HD vs. controls		**Increase** in left (p=0.008) and right (p=0.011) caudate	Right inferior temporal (p=0.011) and right lingual (p=0.007)	NS

Table S4. Network based statistic analysis: Premanifest Huntington's disease vs. control group differences (volume un-normalised) (cortical rich club connections are highlighted in red).

Connection	Test statistic	Connection	Test statistic
R.caudate-R.inferiortemporal	5.89	L.putamen-L.superiorparietal	3.7
L.caudate-R.superiorparietal	5.36	L.caudate-L.postcentral	3.67
L.caudate-L.superiortemporal	5.24	L.caudate-L.putamen	3.67
R.caudate-R.fusiform	4.75	L.caudate-L.lateralorbitofrontal	3.6
L.caudate-L.transversetemporal	4.7	L.caudate-L.entorhinal	3.59
L.caudate-L.precuneus	4.69	R.putamen-L.superiorfrontal	3.55
L.paracentral-R.posteriorcingulate	4.51	L.superiorfrontal-R.paracentral	3.53
R.caudate-R.temporalpole	4.48	R.caudate-R.inferiorparietal	3.52
R.caudate-R.precuneus	4.45	L.bankssts-L.precentral	3.51
L.putamen-L.superiorfrontal	4.28	R.caudate-R.parahippocampal	3.49
R.caudate-R.superiortemporal	4.2	L.thalamus-R.superiorparietal	3.46
L.caudate-L.lingual	4.17	R..caudate-R.parsopercularis	3.44
R.caudate-R.precentral	4.17	L.caudate-L.middletemporal	3.43
L.caudate-L.superiorfrontal	4.16	L.transverse-temporal-L.insula	3.41
L.caudate-L.fusiform	4.12	L.parsopercularis-L.superiortemporal	3.37
R.caudate-R.lingual	4.09	L.caudate-L.bankssts	3.32
R.caudate-R.postcentral	4.03	L.caudate-L.supramarginal	3.29
L.caudate-L.precentral	3.97	R.putamen-R.superiorfrontal	3.28
L.caudate-L.inferiortemporal	3.91	L.caudate-L.superiorparietal	3.26
R.caudate-R.superiorparietal	3.87	L.caudate-L.temporalpole	3.18
R.caudate-R.superiorfrontal	3.79	R.putamen-L.rostralmiddlefrontal	3.18
L.paracentral-R.caudalanteriorcingulate	3.76	L.caudalanteriorcingulate-R.superiorfrontal	3.17
R.putamen-R.caudalanteriorcingulate	3.74	R.caudate-R.isthmuscingulate	3.14
R.paracentral-L.caudalanteriorcingulate	3.74	L.superiortemporal-L.supramarginal	3.12
R.putamen-R.superiorparietal	3.71	L.caudate-R.precuneus	3.11

Table S5. Selective vulnerability: connection density, network traffic (betweenness centrality), regional clustering and distance from the striatum (volume normalised).

Bonferroni corrected $p < 0.05/27$. *Cortico-basal ganglia connectivity analysis.

Volume normalised	HD vs. controls	HD vs. Premanifest HD	Premanifest HD vs. controls
**Streamline density**	Rho = -0.25, p = 1.49 $x10^{-40}$	Rho = -0.45, p = 2.32 $x10^{-142}$	Rho = 0.38, p = 1.45 $x10^{-98}$
**Basal ganglia streamline density**	NS	NS	NS
**Basal ganglia streamline density***	Rho = 0.65, p = 1.05 $x10^{-26}$	Rho = 0.64, p = 2.24 $x10^{-25}$	Rho = 0.45, p = 1.05 $x10^{-11}$
**Betweenness centrality** (degree)	NS	NS	NS
**Betweenness centrality** (strength)	NS	NS	NS
**Clustering coefficient** (degree)	Rho = -0.64, p = 3.35 $x10^{-10}$	Rho = -0.66, p = 7.14 $x10^{-11}$	Rho = -0.37, p = 0.001
**Clustering coefficient** (strength)	NS	NS	NS
**Basal ganglia pathlength**	NS	NS	NS
**Basal ganglia pathlength***	NS	NS	NS
