# Supplementary material for: Early maturation and distinct tau pathology in induced pluripotent stem cell-derived neurons from patients with MAPT mutations
Source: Brain. 2015 Jul 28;138(11):3345–59. doi: 10.1093/brain/awv222 (PMC4620511; doi:10.1093/brain/awv222)
Supplement: Supplementary Fig. 1 [file suppl_data.zip › brain-2015-00254-File014.pdf]

Supplementary Figures

Figure S1. 80-region graph theory analysis: Significant group differences in degree for Huntington's disease vs. controls ($p < 0.0125$). Huntington's disease, premanifest Huntington's disease and controls are presented in each graph to illustrate consistent step-wise reductions in degree across groups. A brain network is displayed above each bar chart. Spheres represent brain regions with red spheres indicating the brain regions showing significance between groups. Data are represented as a group mean (confidence intervals are not included as not standard for permutation tests)

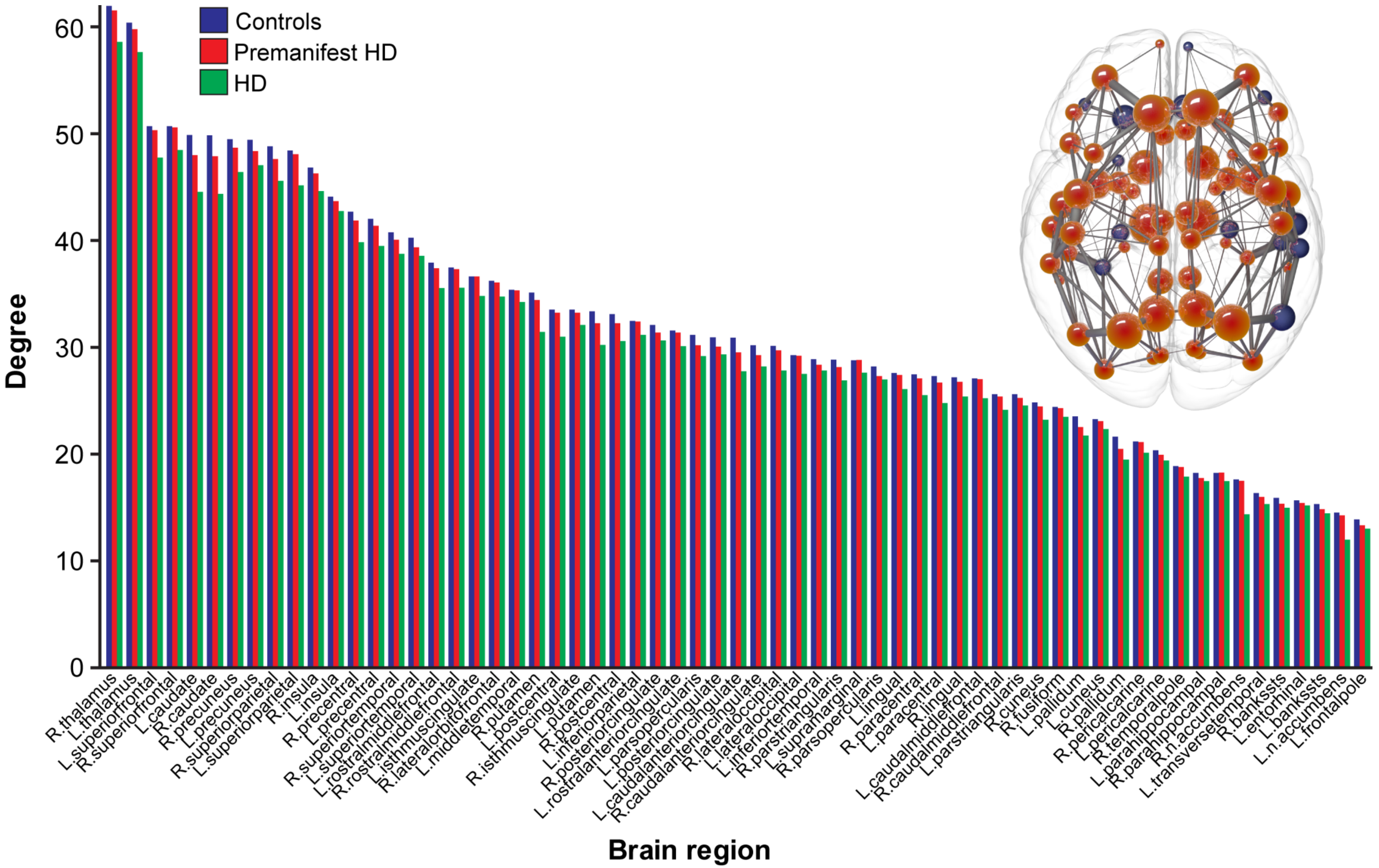
Controls
Premanifest HD
HD
Degree
0
10
20
30
40
50
60
Brain region
R.thalamus
L.thalamus
L.superiorfrontal
R.superiorfrontal
L.caudate
R.caudate
R.precuneus
L.precuneus
R.superiorparietal
L.superiorparietal
R.insula
L.insula
R.precentral
L.precentral
R.superiortemporal
L.superiortemporal
L.rostralmiddlefrontal
R.rostralmiddlefrontal
L.isthmuscingulate
R.lateralorbitofrontal
L.middletemporal
R.putamen
L.postcentral
R.isthmuscingulate
L.putamen
R.postcentral
L.inferiorparietal
R.posteriorcingulate
L.rostralanteriorcingulate
L.parsopercularis
L.posteriorcingulate
L.caudalanteriorcingulate
R.caudalanteriorcingulate
R.lateraloccipital
L.lateraloccipital
L.inferiortemporal
R.parstriangularis
L.supramarginal
R.parsopercularis
L.lingual
R.paracentral
L.paracentral
R.lingual
L.caudalmiddlefrontal
R.caudalmiddlefrontal
L.parstriangularis
R.cuneus
R.fusiform
L.pallidum
L.cuneus
R.pallidum
R.pericalcarine
L.pericalcarine
R.temporalpole
L.parahippocampal
R.parahippocampal
R.n.accumbens
L.transversetemporal
R.bankssts
L.entorhinal
L.bankssts
L.n.accumbens
L.frontalpole

Figure S2. Scatter plots and partial correlations for graph metrics against emotion recognition performance for Huntington's disease, premanifest Huntington's disease and controls. Rho = partial correlation coefficient, DF = degree of freedom.

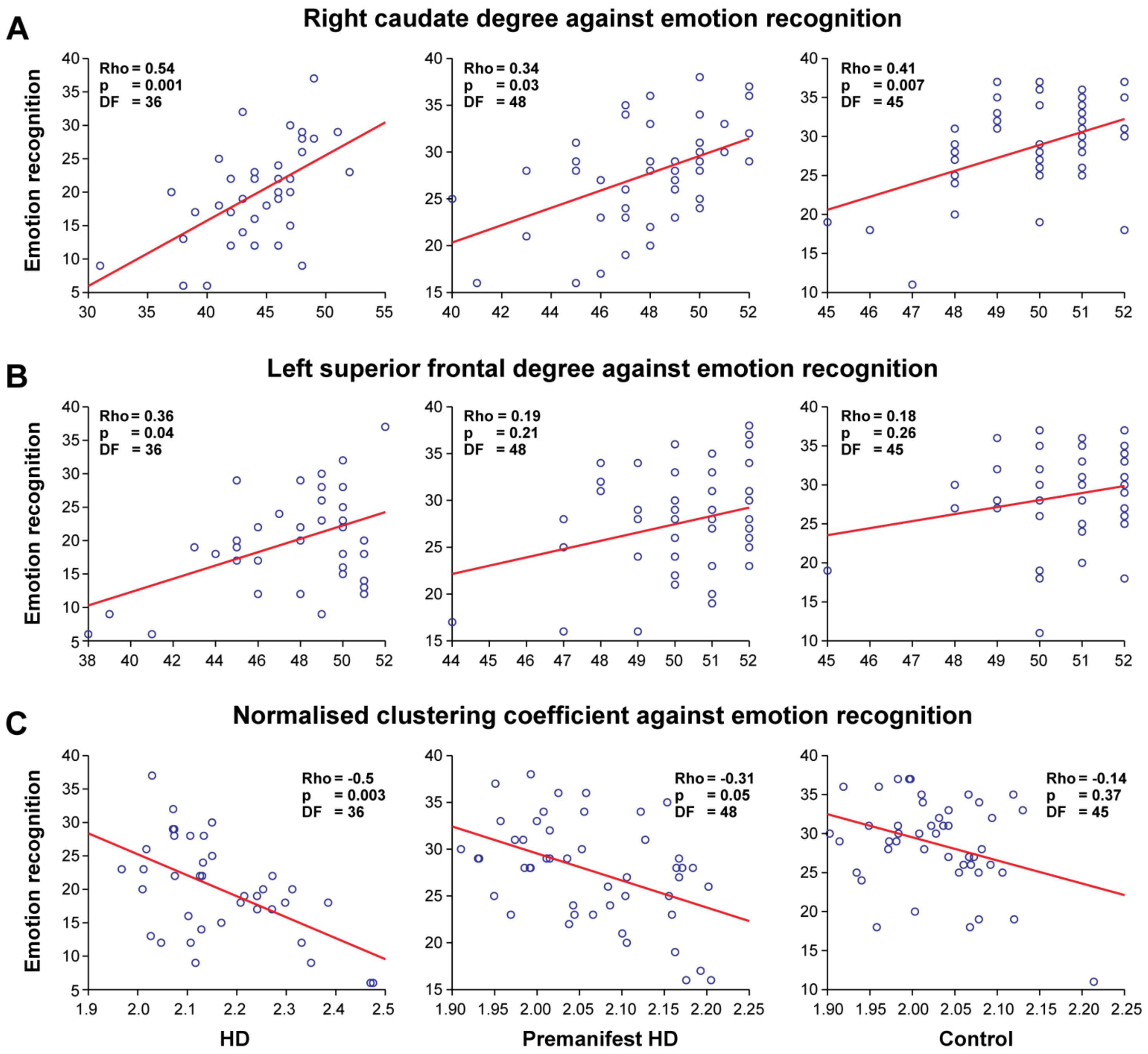

A
Right caudate degree against emotion recognition
Rho = 0.54
p = 0.001
DF = 36
Rho = 0.34
p = 0.03
DF = 48
Rho = 0.41
p = 0.007
DF = 45
B
Left superior frontal degree against emotion recognition
Rho = 0.36
p = 0.04
DF = 36
Rho = 0.19
p = 0.21
DF = 48
Rho = 0.18
p = 0.26
DF = 45
C
Normalised clustering coefficient against emotion recognition
Rho = -0.5
p = 0.003
DF = 36
Rho = -0.31
p = 0.05
DF = 48
Rho = -0.14
p = 0.37
DF = 45
Emotion recognition
HD
Premanifest HD
Control

Figure S3: Cortico-basal ganglia connectivity univariate analysis: Huntington's disease vs. controls (landscape version of 5(c)). Only those connections with $p < 0.002$ are displayed to highlight most significant connections. Data are represented as a group mean (confidence intervals are not included as not standard for permutation tests.

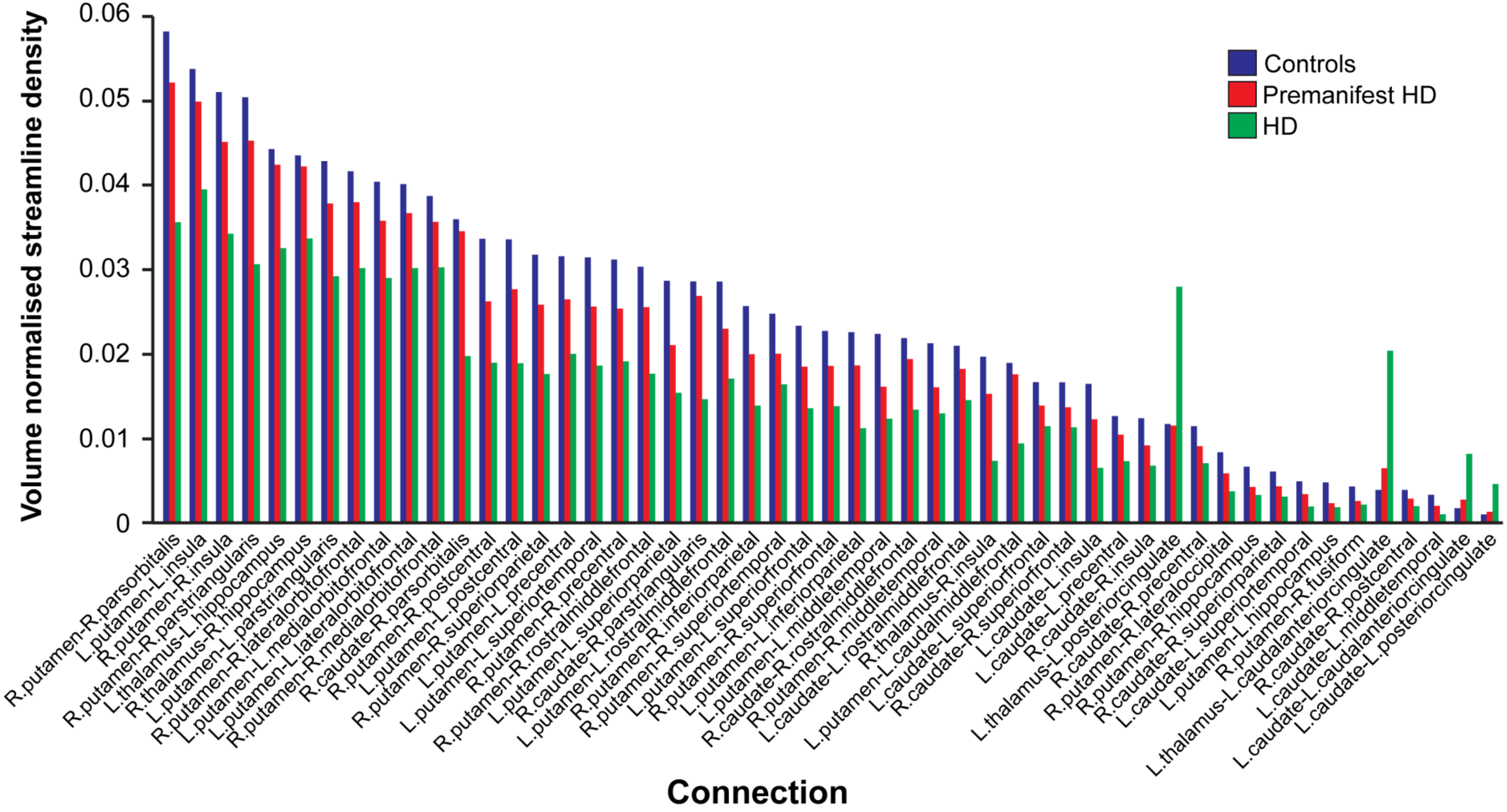
Volume normalised streamline density
0.06
0.05
0.04
0.03
0.02
0.01
0
Controls
Premanifest HD
HD
Connection
R.putamen-R.parsorbitalis
L.putamen-L.insula
R.putamen-R.insula
R.putamen-R.parstriangularis
L.thalamus-L.hippocampus
R.thalamus-R.hippocampus
L.putamen-L.parstriangularis
R.putamen-R.lateralorbitofrontal
L.putamen-L.medialorbitofrontal
L.putamen-L.lateralorbitofrontal
R.putamen-R.medialorbitofrontal
R.caudate-R.parsorbitalis
R.putamen-R.postcentral
L.putamen-L.postcentral
R.putamen-R.superiorparietal
L.putamen-L.precentral
L.putamen-L.superiortemporal
R.putamen-R.precentral
R.putamen-R.rostralmiddlefrontal
L.putamen-L.superiorparietal
R.caudate-R.parstriangularis
L.putamen-L.rostralmiddlefrontal
R.putamen-R.inferiorparietal
R.putamen-R.superiortemporal
L.putamen-L.superiorfrontal
R.putamen-R.superiorfrontal
L.putamen-L.inferiorparietal
L.putamen-L.middletemporal
R.caudate-R.rostralmiddlefrontal
R.putamen-R.middletemporal
L.caudate-L.rostralmiddlefrontal
R.thalamus-R.insula
L.putamen-L.caudalmiddlefrontal
L.caudate-L.superiorfrontal
R.caudate-R.superiorfrontal
L.caudate-L.insula
L.caudate-L.precentral
R.caudate-R.insula
L.thalamus-L.posteriorcingulate
R.caudate-R.precentral
R.putamen-R.lateraloccipital
R.putamen-R.hippocampus
R.caudate-R.superiorparietal
L.caudate-L.superiortemporal
L.putamen-L.hippocampus
R.putamen-R.fusiform
L.thalamus-L.caudalanteriorcingulate
R.caudate-R.postcentral
L.caudate-L.middletemporal
L.caudate-L.caudalanteriorcingulate
L.caudate-L.posteriorcingulate
