# Supplementary figures and images for: Early maturation and distinct tau pathology in induced pluripotent stem cell-derived neurons from patients with MAPT mutations
Source: Brain. 2015 Jul 28;138(11):3345–59. doi: 10.1093/brain/awv222 (PMC4620511; doi:10.1093/brain/awv222)

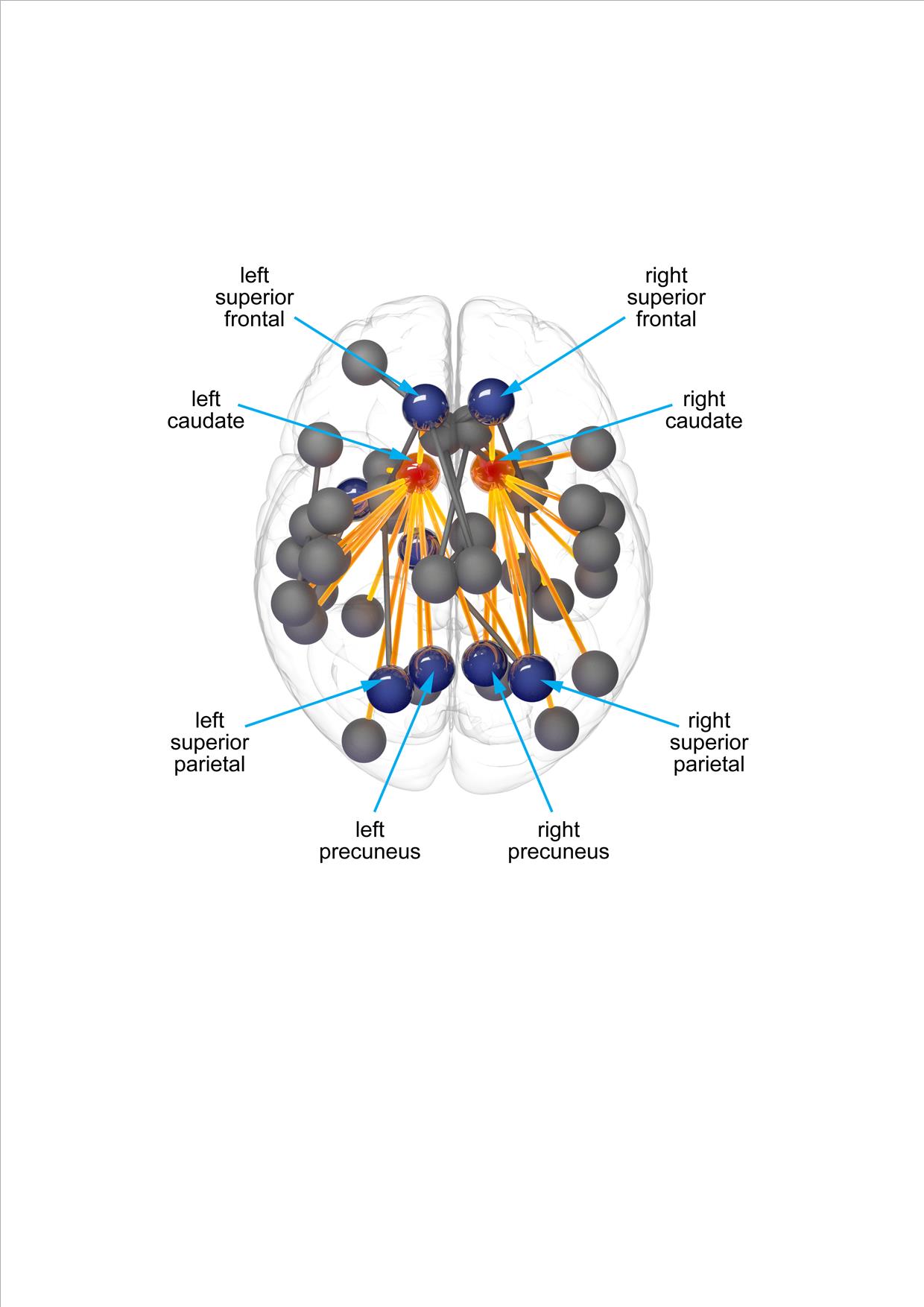

Supplement: Supplementary Fig. 1 [file suppl_data.zip › brain-2015-00254-File011.jpg]
